# Walking in the uncanny valley: importance of the attractiveness on the acceptance of a robot as a working partner

**DOI:** 10.3389/fpsyg.2015.00204

**Published:** 2015-02-25

**Authors:** Matthieu Destephe, Martim Brandao, Tatsuhiro Kishi, Massimiliano Zecca, Kenji Hashimoto, Atsuo Takanishi

**Affiliations:** ^1^Department of Modern Mechanical Engineering, Waseda UniversityTokyo, Japan; ^2^Graduate School of Advanced Science and Engineering, Waseda UniversityTokyo, Japan; ^3^School of Electronic, Electrical and Systems Engineering, Loughborough UniversityLoughborough, UK; ^4^National Centre for Sports and Exercise Medicine – East MidlandsLoughborough, UK; ^5^Leicester-Loughborough Diet, Lifestyle and Physical Activity Biomedical Research Unit, National Institute for Health ResearchLoughborough, UK; ^6^Humanoid Robotics Institute, Waseda UniversityTokyo, Japan

**Keywords:** humanoid robot, emotion, uncanny valley, cross-cultural study, acceptability

## Abstract

The Uncanny valley hypothesis, which tells us that almost-human characteristics in a robot or a device could cause uneasiness in human observers, is an important research theme in the Human Robot Interaction (HRI) field. Yet, that phenomenon is still not well-understood. Many have investigated the external design of humanoid robot faces and bodies but only a few studies have focused on the influence of robot movements on our perception and feelings of the Uncanny valley. Moreover, no research has investigated the possible relation between our uneasiness feeling and whether or not we would accept robots having a job in an office, a hospital or elsewhere. To better understand the Uncanny valley, we explore several factors which might have an influence on our perception of robots, be it related to the subjects, such as culture or attitude toward robots, or related to the robot such as emotions and emotional intensity displayed in its motion. We asked 69 subjects (*N* = 69) to rate the motions of a humanoid robot (*Perceived Humanity, Eeriness*, and *Attractiveness*) and state where they would rather see the robot performing a task. Our results suggest that, among the factors we chose to test, the attitude toward robots is the main influence on the perception of the robot related to the Uncanny valley. Robot occupation acceptability was affected only by *Attractiveness*, mitigating any Uncanny valley effect. We discuss the implications of these findings for the Uncanny valley and the acceptability of a robotic worker in our society.

## Introduction

As Robotics as a science progresses, robots develop improved functionalities. The DARPA (Defense Advanced Research Projects Agency) Robotics Challenge is bringing highly sophisticated robots, mainly humanoids, to the disaster theaters to help humans and assist in rescues. Besides rescuers, humanoid robots may have other roles, especially in our aging society: nurse, receptionist, nanny, house helper, or even kindergarten teacher. When building robots to help or service us, it is important to understand what makes a robot acceptable. For example, the personality of the robot has to adapt to the job itself and not to the users' personality in order to have a higher social trust from them to complete a certain task or job (Joosse et al., 2013). Also, some jobs are favored for robots and some for humans (Takayama et al., 2008). Whenever memorization, acute perception and service to others are the main features of a job description, people would be comfortable to have a robot doing the job. Whenever artistic creation, evaluation, judgment, and diplomacy are required people would prefer a human performing the job.

**Figure 1:**
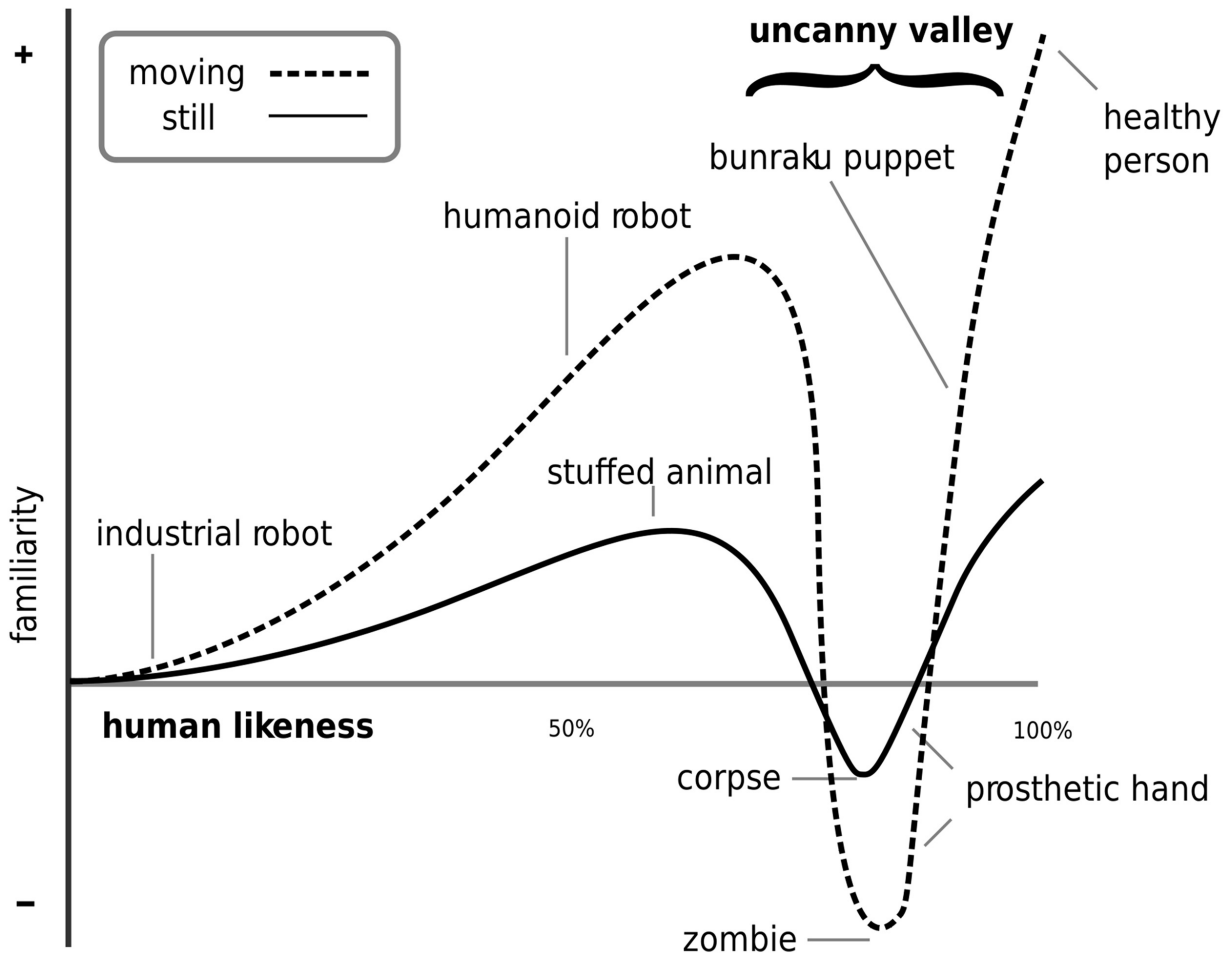
**The Uncanny valley**. Mori describes the Uncanny valley as a non-linear relation between the perceived familiarity felt toward a thing and its human likeness (MacDorman et al., 2005).

Most of the service jobs entail a form of emotion regulation which is called Emotional labor (Hochschild, 2003). Emotion labor jobs require face-to-face interaction with customers and influence their emotional state. In face-to-face interactions, displaying emotions—and sometimes not displaying them or tuning them down—helps the outcomes of said interactions (Dasborough and Ashkanasy, 2002; Prati et al., 2003). For jobs where emotional labor is necessary, would the use of an emotional robot would be adequate (i.e., would the robot transmit the correct message and influence the person it is interacting with in an appropriate way) and more importantly, not provoke feelings of unease? When humanoid robots are designed to interact with people, there is a risk of rejection from the users due to the robots similarity with humans. A hypothesis called “the Uncanny Valley,” quite popular in Human-Robot Interaction (HRI), tries to explain this phenomenon. Developed by Mori in 1970, the Uncanny valley phenomenon occurs when the more human-like a thing is (a doll, a robot, etc.,) the more familiar people feel toward that thing (Mori, 1970). Nonetheless this relationship is not linear: when human-likeness is close to perfect but some differences still exist, the curve collapses and the feeling, which was familiar, becomes uncanny. The term uncanny is the English translation of the German *Unheimlich*, a word describing something being felt simultaneously as familiar, strange, and scary. When the human-likeness reaches the point where it is quite hard to tell the difference from a human being, the curve rises steeply again, outlining the shape of a valley, thus giving the name “Uncanny valley” to that phenomenon (Figure 1) (MacDorman et al., 2005).

Despite its popularity, there is still uncertainty about what would be the cause of this phenomenon. Some recent studies do not support the existence of the Uncanny valley (Bartneck et al., 2009a; Thompson et al., 2011) as they found little to no evidence of the expected results. However, other studies (Ho et al., 2008; Mitchell et al., 2011) support the existence of the phenomenon. Researchers have tried to understand the disparity of the results (Pollick, 2010) but so far no consensus has been reached.

Many studies have been done on the effect of robots' appearance and even some were done on robot movement (Pollick, 2010). Nonetheless the Uncanny valley phenomenon was not studied with humanoid robots expressing emotions with different intensities. As robot developers, we see several limitations in the few studies using robot motions to test the Uncanny valley phenomenon. The first issue is to use of the same movement with different media (human, human-like robot, and machine-like robot) such as performed by Saygin et al. (2012). They discovered that android motions increase brain activity in the action perception system compared to human or robot motions. Nonetheless they found that the repetition suppression effects were stronger for the human-like robot indicating a possible neural basis for the Uncanny valley phenomenon. While this kind of study is interesting *per-se*, it does not inform us about how to improve robot motions and make them more acceptable for users. The second issue is the use of a wide range of different robots performing motions without relation between them (MacDorman, 2006). In the study the author used as stimuli videos of 13 different robots performing diverse activities and found that the humanness of a robot is not the only factor influencing the eeriness perceived by the participants. However, using several robots quite dissimilar in shape and design might hinder the appearance effect and a single (or similar) motion with a neutral meaning should be used to avoid biasing the results. To overcome those limitations, we created several human-like gait patterns with different emotional intensities for a unique full-body human-sized robot and assessed them. Instead of using movements designed by an animator, we use gait data captured from experiments with professional actors (Destephe et al., 2013a). After analysis of the movements, we created for two emotions [Happiness and Sadness two patterns with different intensities (natural and exaggerated emotional intensities)]. We also created a non-emotional pattern to serve as control. Those patterns were assessed by showing videos of the humanoid robot to French and Japanese subjects. They assessed them through a specialized questionnaire (Ho's modified Godspeed questionnaire) (Ho and MacDorman, 2010) and rated their acceptability for different types of jobs.

We propose, in accordance with the Uncanny valley hypothesis, that as the perception of Humanness grows, the Eeriness rating follows an Uncanny valley-like shape. We predict a cultural difference in the perception of the *Eeriness* and *Attractiveness*. Japanese people and French people have a different views on what is natural or artificial (Berque, 1997; Kaplan, 2004). French people see natural things and artificial things as opposed: they see the world as hierarchical, boundaries limiting things and categories defining them. This mindset might be influenced by the Cartesian French education (Weinshall, 1971; Lubatkin et al., 2005). Inheriting a tradition of Buddhist (everything is considered to be a manifestation of same greater concept) and Shintoism (spiritual essence can be manifested in any form from rock to rivers through animals and even humans) (Earhart, 1982), Japanese people would see natural things and artificial things connected and being parts of a bigger picture. These distinctions might influence the *Eeriness* perception: Japanese participants would be less sensitive to discrepancies in the robot, thus rating lower Eeriness than French participants. Japanese people will prefer Natural Intensity emotion representation and French people will prefer Exaggerated Intensity emotion representation. The *Attitude toward robots* and the *Age* factors will predict how people perceive the robot: participants with a positive attitude and young participants (under 30 years old) will rate *Humanness, Attractiveness* higher, and *Eeriness* lower. Participants with a negative attitude and old participants (more than 50 years old) will rate *Humanness* and *Attractiveness* lower, and *Eeriness* higher. Finally, we hypothesize that the perception of the Uncanny valley (robot being eerie or not) will influence the participants to say whether an occupation is acceptable or not for the robot.

## Methods

### Participants

A total of 70 subjects participated to this experiment but one was excluded from the analysis due to a software issue (*N* = 69). The participants were invited to participate to a study about HRI through announcement in class, social network services, and mailing-lists. This study is a cross-cultural study between French and Japanese people. A total of 47 French subjects participated to this experiment (*N*_FR_ = 47) with an average age of 34.7 ± 12.5 years old ranging from 21 to 81 years old [28 males (33.9 y.o. ± 12.5) and 19 females (35.9 y.o. ± 12.6)]. A total of 22 Japanese subjects participated to this experiment (*N*_JP_ = 22) with an average age of 29.2 ± 7.1 years old ranging from 21 to 53 years old [9 males (26.3 y.o. ± 5.0) and 13 females (32.2 y.o. ± 7.9)]. The ethical committee approved the experiment protocols, the participants gave us their written informed consent and all the data collected are anonymized. The participants were recruited through on social network websites, general forums, and mailing-lists with no relation to robotics or robots.

### Our robot

**Figure 2:**
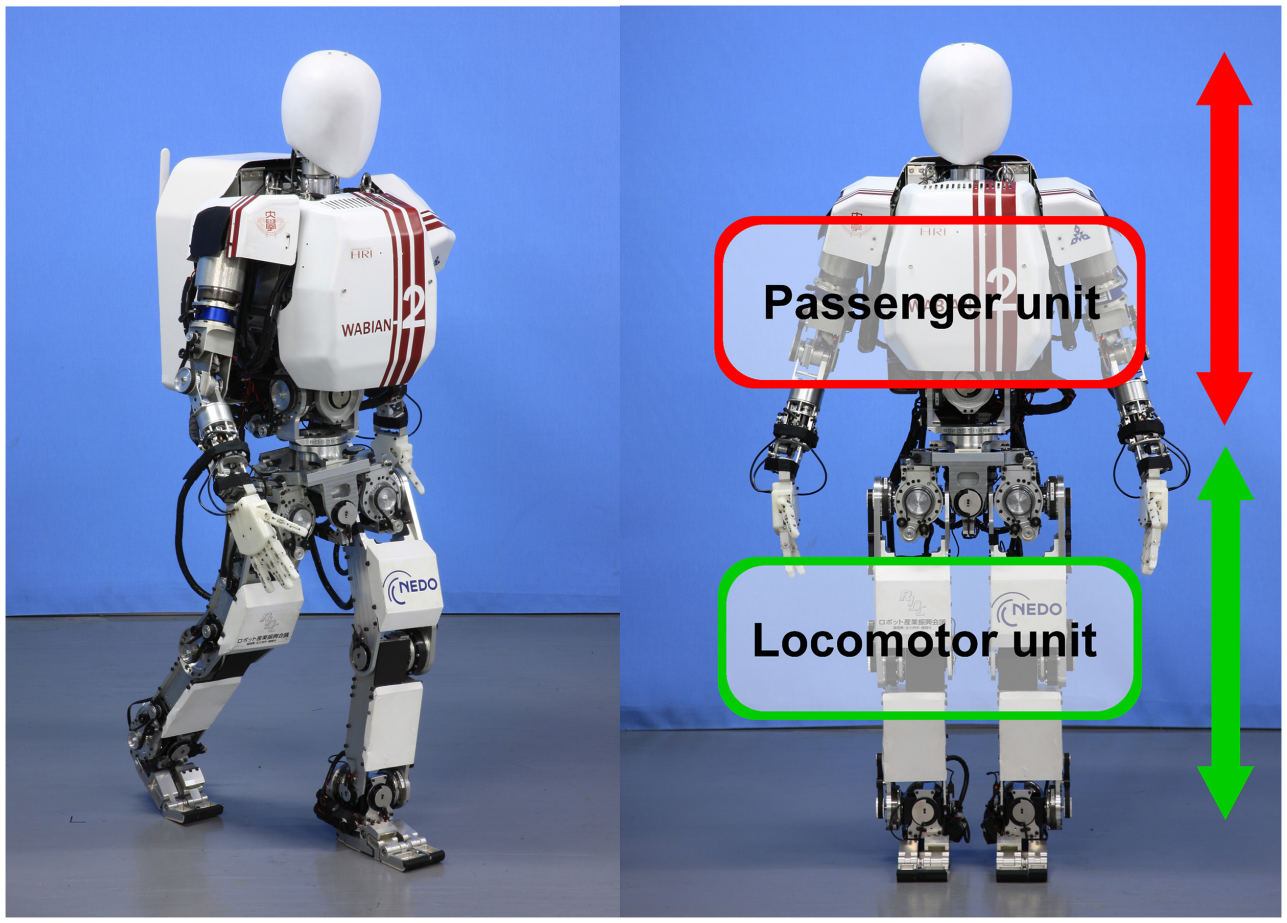
**The humanoid robot WABIAN-2R**. It is capable of human-like walking with its stretched knees and pelvis mechanism.

The videos used for our work are based on the humanoid robot WABIAN-2R (Figure 2). Unlike most bipedal humanoid robots, WABIAN-2R is able to perform a human-like walking with stretched knees thanks to its 2-DoF waist during the stance phase while other robots walk with bent knees (Ogura et al., 2006). WABIAN-2R is 1.5 m in height, and 64 kg in weight. Its design allows human-like gait including heel-contact and toe-off phases. This robot is mainly used for locomotion experiments and to study human movements. Besides an advanced locomotion technology, the head is a neutral, stylized human-like shape with no distinguishable features. We chose this robot because having no facial expression helps to focus on the expressivity of the whole body without having any influence coming from facial expressions.

### Stimuli

The robot emotional walking patterns were created from our previous study (Destephe et al., 2013a). Two professional actors (who acted in plays, drama, and movies) were asked to perform several types of emotional walking such as Sadness, Happiness, Anger, and Fear and with different intensities: Natural (Low, Intermediate, and High) and Exaggerated. We categorize the three intensities (Low, Intermediate, and High) as Natural because the actors were asked to act in such a way that they would correspond to natural occurrences of emotion expression in daily life. The Exaggerated intensity on the other hand were performed with extravagant theatricality, broad gestures, and overplayed expressions, comparable to emotions expressions seen in plays and theaters.

**Figure 3:**
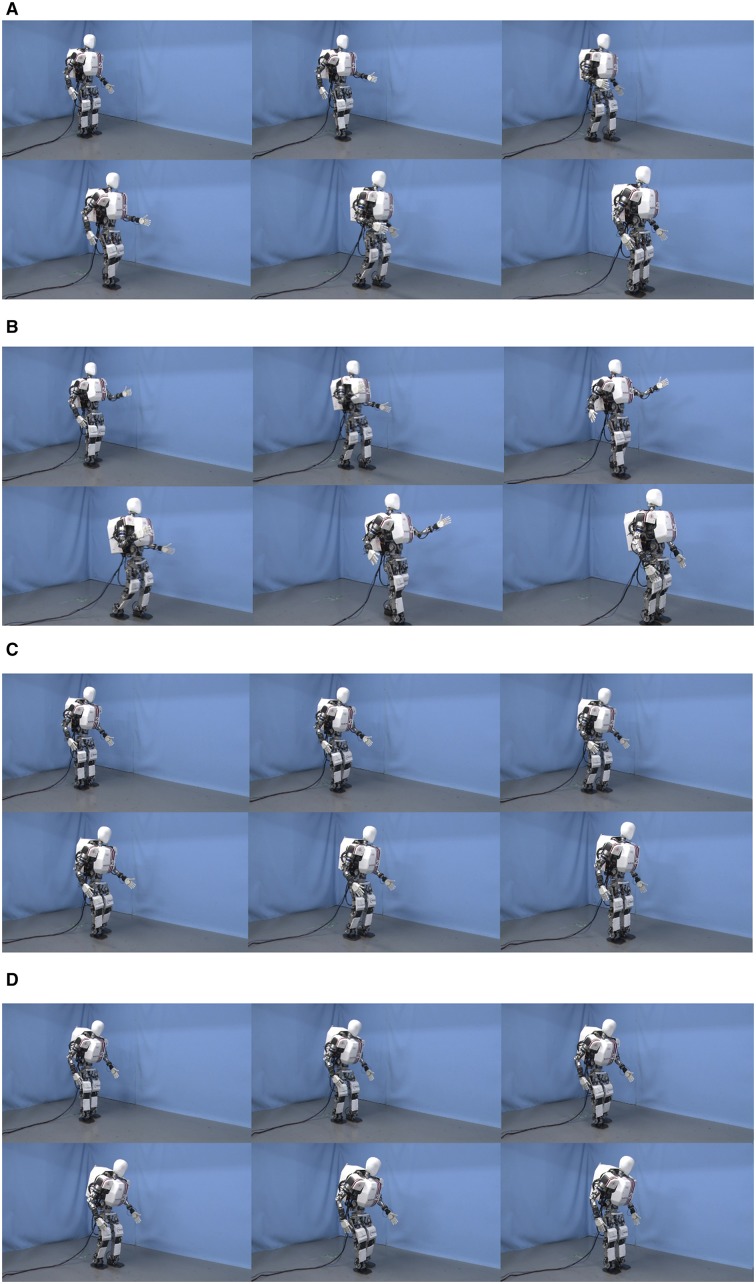
**Emotional gait patterns**. The following images are captured from the videos shown to the participants. **(A)** represents Happy walk (Natural intensity); **(B)** Happy walk (Exaggerated intensity); **(C)** Sadness (Natural intensity); **(D)** Sad walk (Exaggerated intensity).

For this work, we used Happiness and Sadness walking patterns and for each of them, one walking pattern of Natural intensity (High) and another of Exaggerated intensity. The walking patterns were based on actors' whole body movements (Destephe et al., 2013a). They were created manually such as to approximate the actors' motion as much as possible, within the constraints related to differences in the structure and the dynamics of a human body and a humanoid body. We scaled the actors' values to respect the hardware limits of the robot and used our pattern generator to generate stable walking patterns. In our previous work (Destephe et al., 2013b), the gait patterns we created achieved a high recognition rate (Natural (High) intensity/Exaggerated intensity) (Happiness: 75.0/85.7%; Sadness: 75.0/92.9%) when we assessed them in simulation with subjects. Examples of patterns used in this study are shown in the Figure 3.

### Questionnaire

The questionnaire we gave to the participants of this study is composed of four sub-questionnaires. For a description of the complete questionnaire, please refer to the reproduction given in the Supplementary Material.

**Sub-questionnaire #1**. The first sub-questionnaire asks for general information: sex, age, nationality, education level and current occupation.
**Sub-questionnaire #2**. The second sub-questionnaire enquires about the participant's robot-related experiences and their attitude toward robots based on the MacDorman questionnaire (MacDorman et al., 2008).
**Sub-questionnaire #3**. The third sub-questionnaire is described as a personality questionnaire. In fact, that questionnaire is a short screening questionnaire for autism called AQ10 (Autism spectrum Quotient with 10 items) (Allison et al., 2012).
**Sub-questionnaire #4**. The last sub-questionnaire assesses the participant's reactions and feelings about our emotional robot and is based on Ho's questionnaire (Ho and MacDorman, 2010). This questionnaire is designed to assess if there is the Uncanny valley phenomenon. There are several popular questionnaires used to study the reaction of robots' users such as the Godpseed questionnaire (Bartneck et al., 2009b). The Godspeed questionnaire is not well-adapted to measure the reactions to humanoid robots as several scales are redundant and it does not evaluate well the Uncanny valley phenomenon. Two issues are occurring with the Godspeed questionnaire. First, some of the semantic items do not encode well enough the indices they are related to. Second, Anthropomorphism, Likeability, Animacy, and Perceived Intelligence are highly correlated between themselves, therefore they encode the same concept (most probably the humanness of the study subject) instead of encoding for different concepts (Ho and MacDorman, 2010). This questionnaire is made of four different pages. The first page shows a video of the real robot walking without emotion (normal walking) and a text stating “This is the normal emotionless walking robot. Please watch it walking. Normal walking (without emotion)” located at the top of the video. The second, third and fourth page have the same layout. First, a video of the robot walking with an emotion is displayed. Then, the participant is asked the two following questions: (i) “What do you think the robot expressed as emotion?”; (ii)“In what kind of environment and place the movements and the emotions of the robot would be the most relevant?”

This questionnaire was conducted online. The videos used as stimuli lasted between 5 and 10 s and were provided with sound (mainly robot actuators were audible). The participants were given the possibility to replay them at will. The participants were asked to rate the robot and its walking. The questionnaire measures three categories: *Perceived Humanness, Eeriness*, and *Attractiveness*. *Perceived Humanness* represents the degree of humanity and human-like characteristics in the robot tested. The *Eeriness* describes the feeling of strangeness, disgust, and familiarity occurring at the same time when something seems natural but some details are not quite conform to the expectation. The *Attractiveness* characterizes the level of comfort and physical attraction we might feel by looking at the robot.

**Table:** 

Perceived Humanness: What do you think about the movements of the robot?						
Artificial	1	2	3	4	5	Natural
Synthetic	1	2	3	4	5	Real
Inanimate	1	2	3	4	5	Living
Human-made	1	2	3	4	5	Humanlike
Mechanical Movement	1	2	3	4	5	Biological Movement
Without Definite	1	2	3	4	5	Mortal
Lifespan

**Table:** 

Eeriness: What are your feelings about the robot?						
Reassuring	1	2	3	4	5	Eerie
Numbing	1	2	3	4	5	Freaky
Ordinary	1	2	3	4	5	Supernatural
Uninspiring	1	2	3	4	5	Spine-tingling
Boring	1	2	3	4	5	Thrilling
Predictable	1	2	3	4	5	Mortal
Bland	1	2	3	4	5	Uncanny
Unemotional	1	2	3	4	5	Hair-raising

**Table:** 

Attractiveness: What do you think of the robot's appearance?						
Unattractive	1	2	3	4	5	Attractive
Ugly	1	2	3	4	5	Beautiful
Repulsive	1	2	3	4	5	Agreeable
Crude	1	2	3	4	5	Stylish
Messy	1	2	3	4	5	Sleek

All fields were mandatory. A total of two emotional gaits (Happiness and Sadness) and a neutral gait (for reference and stated as neutral gait) were shown randomly to each participant. For a given participant the intensity of the emotional gaits was fixed: Natural (High) or Exaggerated. For the French people, 23 subjects were randomly exposed to the Natural (High) intensity and 24 to the Exaggerated intensity. For Japanese people, 11 subjects were randomly exposed to the Natural (High) intensity and 11 to the Exaggerated intensity. The results regarding the Autism questionnaire are not discussed in this work.

### Results

#### Attitude toward robots

First we want to explore the pre-conceived ideas about robots for the different factors we are considering for our study. All the participants were not only divided by nationality (French or Japanese) but were also divided according to their *attitude toward robots* (positive or negative), their *age* (young age, middle age or old age), their *familiarity with robots* (not familiar or familiar), and their *interest for robots* (not interested or interested) (Table 1). The values are on a scale between −3 and +3 (7-Likert scale), with negative values indicating a disagreement with the item and positive values agreeing with it. *Exposure to robots* indicates how many exposures had the participant with robots through media, events, programming, etc. *Robot preference* shows whether the participant prefer people (reported as negative value) or robot (reported as positive value). *Warmness toward robots* points out whether the participant is cold (reported as negative value) or warm toward robots (reported as positive value). *Warmness toward people* reports whether the participant is cold (reported as negative value) or warm toward people (reported as positive value). *Robots' threat* informs about the participant's feelings on whether robots are more threatening than people (reported as negative value) or the inverse (reported as positive value). *Robots are safe* indicates whether the participant feels that robots are threatening (reported as negative value) or safe (reported as positive value) and *People are safe* whether the participant feels that people are threatening (reported as negative value) or safe (reported as positive value). We performed a multi-factorial ANOVA to examine the effects of the different factors (culture, general attitude toward robots, etc.) on the attitudes (exposure to robots, robot preference, etc.).

**Table 1:**
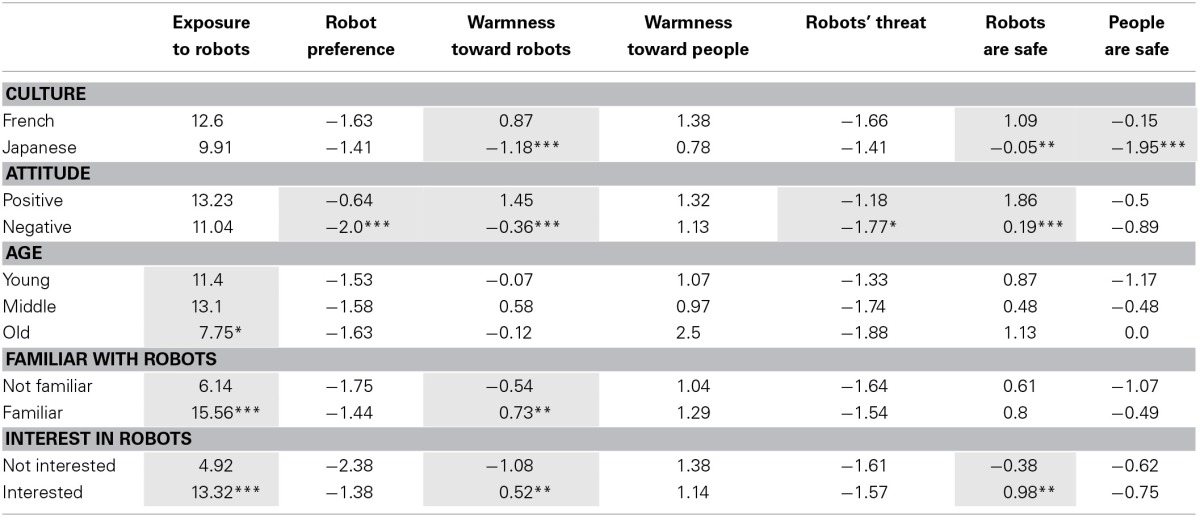
**Attitudes toward robot and people per factor**.

The statistical significance is shown in grayed table cells. (^***^p < 0.001; ^**^p < 0.01; ^*^p < 0.05).

***Culture***. Contrary with the common stereotype depicting Japanese people as people quite fond of robots, French participants feel warmer toward robots than Japanese participants [*F*_(1, 50)_ = 34.966, *p* < 0.001] and feel also safer with them [*F*_(1, 50)_ = 11.428, *p* < 0.01]. Japanese participants tend to find people not safe [*F*_(1, 50)_ = 47.594, *p* < 0.001] while French participants are rather moderate. Both cultures prefer, on average, people to robots but trust more robots than people.

***Attitude toward robots***. The attitude toward the robots is determined by calculating the mean of the “Prefer robots,” “Warm toward robot,” “Robot are more threatening,” “Robots are safe” items. If the value is less than 0, the attitude is negative; and more than 0, the attitude is positive. While positive-minded participants like equally people and robots, negative-minded participants clearly prefer people to robots [*F*_(1, 50)_ = 28.614, *p* < 0.001]. Similarly, positive-minded participants would feel warmer toward robots [*F*_(1, 50)_ = 11.892, *p* < 0.01] and also safer with them [*F*_(1, 50)_ = 16.854, *p* < 0.001] while negative-minded participants are more reserved and find that robots are much more a threat than positive-minded participants [*F*_(1, 50)_ = 4.596, *p* < 0.05].

***Age***. The age category is divided in three: young (under 30), middle-aged (between 30 and 50) and old (more than 50). Old participants were less exposed to robots than the younger articipants [*F*_(2, 50)_ = 3.505, *p* < 0.05].

***Familiarity***. Participants considering themselves familiar with robots have more exposures to them than non-familiar participants [*F*_(1, 50)_ = 38.982, *p* < 0.001] and tend also to be warmer toward robots [*F*_(1, 50)_ = 5.198, *p* < 0.05].

***Interest***. Interested participants have more exposures to robots [*F*_(1, 50)_ = 42.832, *p* < 0.001], are warmer to robots [*F*_(1, 50)_ = 10.629, *p* < 0.01] and find robots rather safe [*F*_(1, 50)_ = 7.957, *p* < 0.01] than the non-interested participants.

We performed a Kolmogorov-Smirnov test and found a statistical difference between the warmth felt toward a robot (*M* = 0.19; *SD* = 0.89) or a human (*M* = 1.26; *SD* = 0.47) [*D*_(11)_ = 0.75, *p* < 0.01]. Regarding safety, participants clearly favor robots (*M* = 0.78; *SD* = 0.68) over humans (*M* = −0.68; *SD* = 0.54) [*D*_(11)_ = 0.8333, *p* < 0.001].

#### The Uncanny valley

We chose to use the Ho questionnaire which is a modified version of the Godpseed questionnaire to evaluate reactions regarding the Uncanny valley. That questionnaire was designed to test three different groups of items: *Perceived Humanness, Eeriness*, and *Attractiveness*, rated from 1 (low) to 5 (high). We tested the whole questionnaire results for reliability: *Perceived Humanness* (Cronbach's α: 0.77), *Eeriness* (Cronbach's α: 0.85), and *Attractiveness* (Cronbach's α: 0.84). Therefore, the questionnaire has a good reliability.

By using the Ho questionnaire, we measure the possible differences existing in the perception of emotional movements and their link to the Uncanny valley phenomenon. We also investigate if several factors would influence the perception such as the emotional intensity, the type of emotions, the culture of the participants, their attitude toward robots, their age, their familiarity, and their interest.

To begin, we analyze the possible difference in the recognition of the emotions between the cultural groups (Table 2). We used Pearson's Chi-squared Test in order to compare the two groups. We found no statistical difference between the two groups (French: 51.1%, Japanese: 63.3%, χ^2^ = 1.4403, *p* > 0.05). The recognition rate for each emotion was not significantly different between the groups: Happiness (French: 42.6%; Japanese: 59.1%, χ^2^ = 1.0466, *p* > 0.05), Sadness (French: 59.2%; Japanese: 68.2%, χ^2^ = 1.1773, *p* > 0.05). We confirm that the participants performed above chance level (20%) (χ^2^ = 103.9135, *p* < 0.000).

**Table 2:** **Emotion recognition**.

**Emotions**	**French**	**Japanese**
Happiness	42.6%	59.1%
Sadness	59.2%	68.2%
Average	51.1%	63.3%

#### Is it a valley?

**Figure 4:**
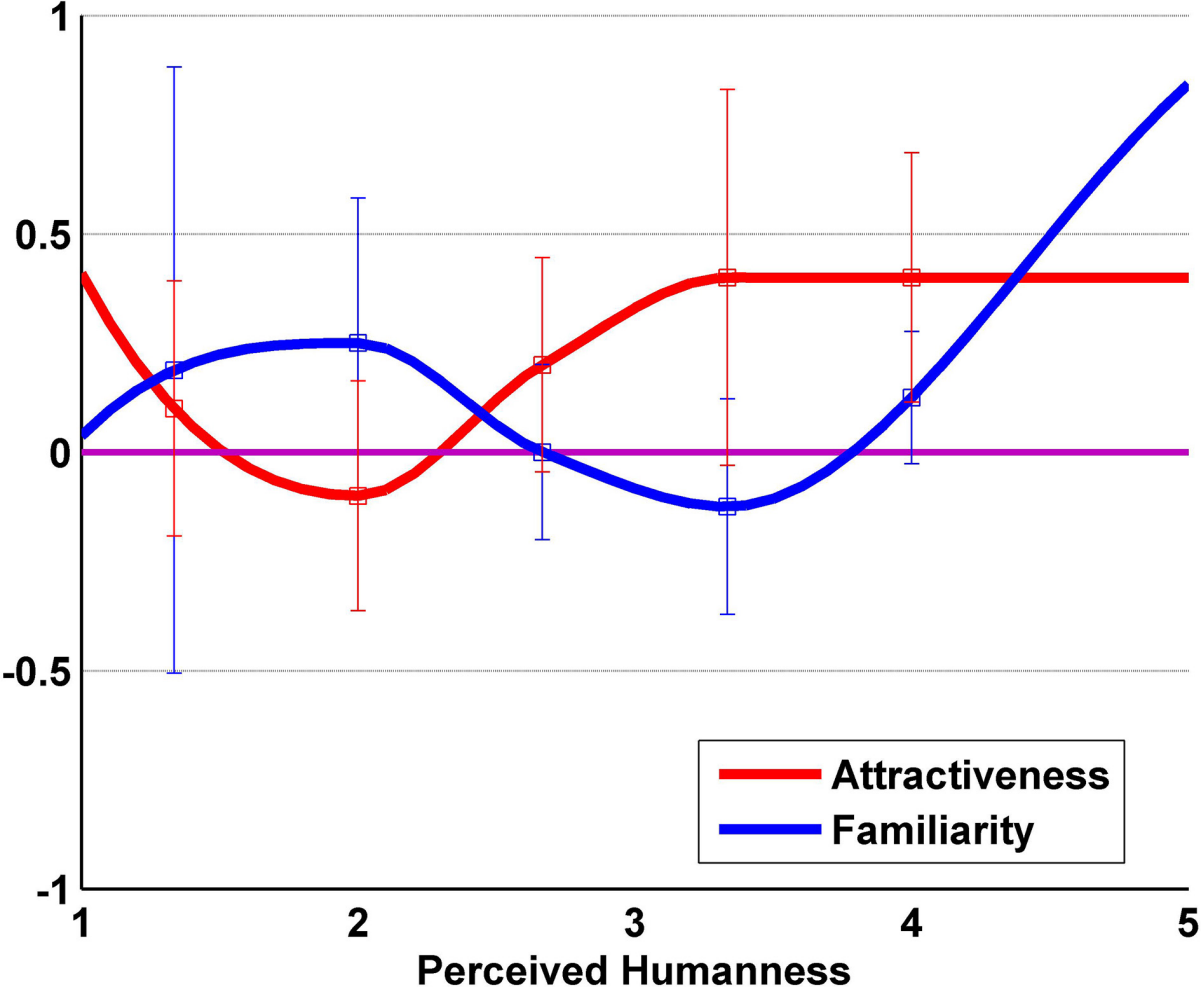
**Our Uncanny valley**. Perceived Humanness and Familiarity (reverse scaled Eeriness) are plotted in order to describe the Uncanny valley. Attractiveness is also plotted as reference.

According to Ho et al. (Ho and MacDorman, 2010), the *Eeriness* and *Perceived Humanness* can be plotted together to obtain a graph similar to Mori's Uncanny Valley figure. Nonetheless, the *Eeriness* values have to be transformed into *Familiarity* values by reversing the 5-Likert Type scale (1 becomes 3 and 5 becomes −3) and center the values around 0. We plot *Attractiveness* and *Familiarity* scores against the *Perceived Humanness* score (Figure 4). From the plot, we observe two interesting results: an Uncanny valley-like curve for the *Familiarity* score and an another valley-like curve for the *Attractiveness* score.

#### Cultural difference

As the data we want to analyze are unbalanced and the variables are both categorical and continuous, we use a Generalized Linear Model (GLM) to test each questionnaire item group (*Perceived Humanness, Eeriness*, and *Attractiveness*) with Culture (*French* vs. *Japanese*), Emotion (*Happiness* vs. *Sadness*), and Intensity (*Natural* vs. *Exaggerated*) as independent variables. We found that Intensity is a main effect for *Perceived Humanness* item [*F*_(1, 130)_ = 11.943, *p* < 0.001] (Low: 2.42 ± 0.86; High: 2.88 ± 0.69) and the *Nationality* is a main effect for the *Attractiveness* item (*French*: 3.18 ± 0.6; *Japanese*: 2.97 ± 0.5). We tested further the within condition Intensity for French and Japanese participants. The *Intensity* condition only affected the *Attractiveness* felt by Japanese participants [*F*_(1, 42)_ = 4.172, *p* < 0.05; *Low*: 3.12 ± 0.5; *High*: 2.81 ± 0.5]. In summary, Japanese people prefer (higher score of *Attractiveness*) Natural Intensity emotions feelings over Exaggerated Intensity emotions and French people prefer neither Natural nor over Exaggerated Intensity emotions.

#### Attitudes and other factors

We found that the *Attitude toward robots* has a main effect on *Eeriness* and *Attractiveness* questionnaire items. The *Exposure to robots* has also a main effect on the *Attractiveness*. The *Age* × *Attitude* interaction showed to have an significant effect on *Perceived Humanness* and *Attractiveness*. For *Perceived Humanness* we found the following interactions: *Interest* × *Exposures*, *Interest* × *Familiarity*, and *Familiarity* × *Exposures*. For *Attractiveness* we found the following interactions: *Age* × *Familiarity*, *Age* × *Exposures, Attitude* × *Exposures*, and *Familiarity* × Attitude. All the statistical results are presented in Table 3. To summarize, *Attitude toward Robots* is the main predictor for the *Eeriness* and *Attractiveness* items with the *Exposures to robots* being closely related to it, i.e., if you like robots you will try to be more exposed to them.

**Table 3:**
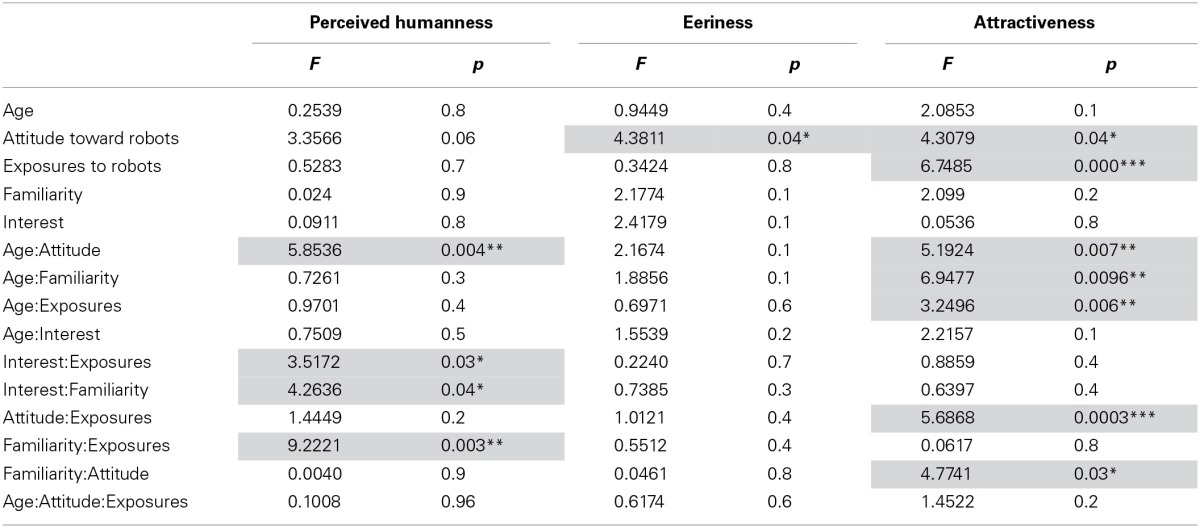
**Attitudes and other factors influences on the perception of the robot**.

The statistical significance is shown in grayed table cells. (^***^p < 0.001; ^**^p < 0.01; ^*^p < 0.05).

#### Occupation acceptability

**Figure 5:**
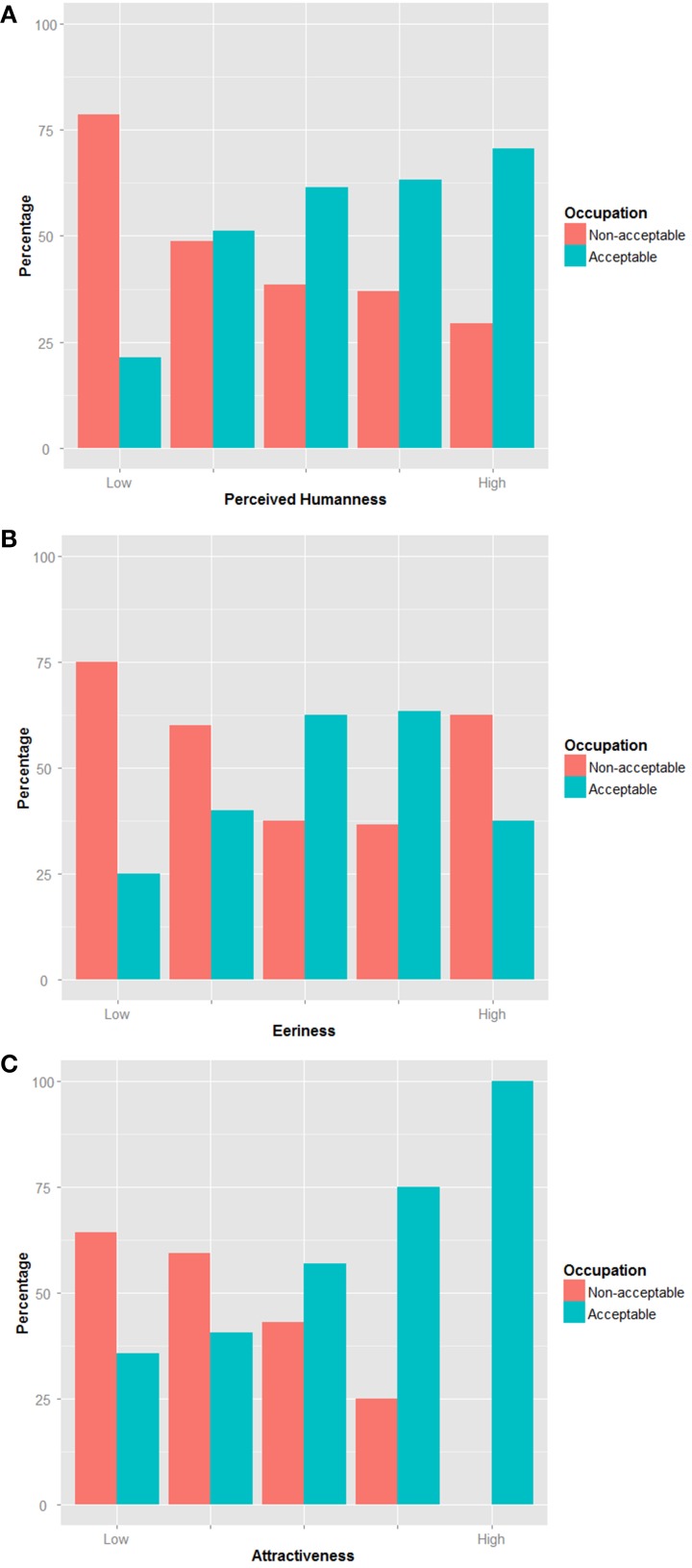
**Robot occupation acceptability**. The images represent the occupation acceptability of related to the participants' ratings of **(A)** Perceived Humanness; **(B)** Eeriness; **(C)** Attractiveness.

We have categorized the occupations in two groups: *Acceptable* and *Non-acceptable* occupation. The *Acceptable* group consists of Police, School, Office, and Hospital related occupations answers and the *Non-acceptable* group of Nowhere answers. We performed an analysis of the correlation using Spearman method to understand how the Occupation acceptability would be influenced by the *Perceived Humanness, Eeriness*, and *Attractiveness* felt by the participant. The analysis yielded the following results: *Perceived Humanness* (*r_s_* = 0.209, *p* < 0.05) and *Attractiveness* (*r_s_* = 0.347, *p* < 0.000). The correlation coefficients suggest that *Attractiveness* is a good predictor (medium effect size) of the Occupation acceptability and *Perceived Humanness* also affects it (small effect size). *Eeriness* ratings did not affect the participants' view on the robot occupation acceptability (not significant) (Figure 5). This means that the perception of the Uncanny valley (*Eeriness*) does not affect the acceptability of the robot for a given job and the perceived *Attractiveness* will mostly affect its acceptance, followed by *Perceived Humanness*.

## Discussion

First we wanted to investigate factors (cultural background, attitude toward robots, age, interest in robots and familiarity with them), and how those factors might influence our perception of the robot and the Uncanny valley. Then, from those observations, we analyzed their effects on the Uncanny valley phenomenon. Finally, we examined the impact of the participants' perception on the acceptability of the robot's possible occupation.

### Attitude toward robots

#### Culture

Bartneck and MacDorman studied how people view robots (Bartneck et al., 2007a; MacDorman et al., 2009). Bartneck et al. did a cross-cultural study on people's attitude related to robots. US participants were the most positive toward robots while Mexicans were the most negative toward them (the sample size was small, so the results might biased for Mexicans). He remarked that the Japanese were not as fond as the media seems to portray. MacDorman et al. focused his work on the difference between American and Japanese faculty and also provided proofs against the common stereotype about the Japanese craze for robots. They report no tangible difference observed between American and Japanese faculty. Our results, while focusing on French and Japanese people, support the findings of the two previous studies by Bartneck and MacDorman. Compared to Japanese participants, French participants felt warmer to robots and feel also safer with them. A survey (European Commission, 2012) from the European Union about the attitude toward robots of European citizens reports that 70% (67% for French citizens) of them have a positive view on robots, which tend to support our findings. Japanese participants tended to find people not safe while French participants were rather moderate in that aspect. Both cultures prefer, on average, people to humans but trust more robots than people for their safety.

#### Attitude toward robots

The main effects of the participants' attitude were on their preference between robots and people, the warmness of their feelings for robots and the sense of security they would feel with them. More interestingly, negative-minded participants were not rejecting robots and were rather moderated in their feelings when it comes to robots.

#### Age

Mitzer et al. found that elderly people have a rather positive attitude regarding assistive technology (Mitzner et al., 2010). Scopelliti et al. on the other hand reported contrary results stating that elderly people express mistrust in technology in general (Scopelliti et al., 2005). When focusing on robots, they noticed that young people rated higher positive feelings and elderly people were the most fearful about robots. While we found that old participants (over 50 years old) feel quite warm toward people and trust people more than the other age categories, the age did not seem to affect the participants' view of the robots. Kuo et al. investigated the influence of age on the attitude toward robots and did not find difference between younger people and elderly people (Kuo et al., 2009) which support our results.

#### Familiarity and interest

Both participants considering themselves familiar with robots or interested in them have more exposures to them and feel warmer to them too than their non-familiar or non-interested counterparts. Furthermore interested participants prefer robots and find robots safer than the non-interested participants. According to the familiarity principle (the more you are exposed to a thing or a person, the more likeable it will appear to you) (Bornstein, 1989), exposure to robots is most likely the cause explaining the positive view of the robot of the familiar and interested participants.

Finally, we notice that over the five different factors there is no extreme reject of robots. There is a general trend among the participants: they feel closer to other people but they feel safer with robots. As humans, we naturally feel close to beings that look like us and behave like us, especially when we have to choose between organic and inorganic beings. We associate robots with order, logic, and efficiency. Usually represented with a lack of intent, we see robots as predictable beings therefore they might appear safer and less prone to errors than other human fellows. This feeling of safety is important to understand if we want to bring more robotic workers in our society (Takayama et al., 2008). For example, if we consider robots working in fully automated factories the feeling of safety is not mandatory as we will not interact with them. However, for robots in contact with people such as security workers, healthcare helpers, education assistants, and so on. this safety feeling might be influential in the acceptance of a robotic worker.

### The uncanny valley

#### The valley

Some researchers suggested that the Uncanny valley is rather a cliff than a valley (Bartneck et al., 2007b). Their conclusions are drawn from the mapping of the Likeability in the Human-likeness space and fit the data with a quadratic curve. The claim for an Uncanny cliff instead of an Uncanny valley seems to be overstretch in this context. We formulated the hypothesis that our results would describe a valley-like shape similar to the Uncanny valley hypothesis (Figure 4). Our results show a similarity with the Uncanny valley hypothesis figure (Figure 1) which supports our hypothesis. Tung et al. investigate children's attitude toward robots with two conditions: static and moving (Tung and Chang, 2013). Their results show that the static condition supports well the Uncanny valley static curve. Furthermore, contrary to what is hypothesized in Mori's Uncanny valley hypothesis, the moving condition seems to mitigate the effects of the Uncanny valley instead of amplifying them. In our results, the *Familiarity* values range between a minimum value of −0.12 and a maximum value of 0.84. Those values seem to support the mitigation effect of the motions on the Uncanny valley phenomenon found by Tung et al.

The robot whether perceived totally not human-like (1 on *Perceived Humanness* scale) or fairly human-like to quite human-like (slightly over 3–5 on *Perceived Humanness* scale) is seen similarly attractive to the participants. This would suggest that people would prefer a robot whom they perceive either as quite robot-like or a quite human-like in appearance and behavior. The in-between would be looked down especially if the robot would appear more robot-like than human-like. While we conducted our experiment with only one humanoid robot, we expect this result to be observable in other humanoid robots expressing human-like behavior.

#### The culture and emotions

We predicted that the culture of the participants will influence in the perception of the *Eeriness* and *Attractiveness*. After analysis, Japanese people were found to prefer (higher score of *Attractiveness*) Natural Intensity emotions feelings over Exaggerated Intensity emotions. Contrary to our expectations, this was the only difference we found regarding the influence of Intensity. This difference may be explained by the Japanese perception of acceptable display of emotions. Several cultures such as Korea and Japan expect neutral display of emotions or low intensity emotions (Trompenaars, 1996). Trompenaars did a cross-cultural study including French and Japanese managers. He found that 42% of the Japanese participants think the emotions should not be displayed overtly and only 14% of French participants think likewise which support our findings. Furthermore, the results from the questionnaire about the participants' attitude toward robots also corroborate that French people have warmer feelings for robots than Japanese people thus explaining the influence of the Culture on the *Attractiveness*.

#### The attitude and other factors

We predicted that *Attitude toward robots* would influence how people perceive the robot. Positive attitude will rate *Humanness* and *Attractiveness* higher, and *Eeriness* lower and Negative attitude will rate *Humanness* and *Attractiveness* lower, and Eeriness Higher. Our results only confirm that *Attitude toward robots* affects *Eeriness* (Positive: 2.79 ± 0.58; Negative: 2.99 ± 0.54) and *Attractiveness* (Positive: 3.21 ± 0.66; Negative: 3.06 ± 0.54). While the *Age* factor was present in several interactions, it was not by itself a good predictor for any of *Humanness, Eeriness*, or *Attractiveness*. In a recent study, MacDorman and Entezari propose to examine nine individual differences (Perfectionism, Neuroticism and Anxiety, Animal Reminder Sensitivity, Personal Distress, Human–Robot Uniqueness, Human–Android Uniqueness, Religious Fundamentalism, and Negative Attitudes Toward Robots) (MacDorman and Entezari, 2015). Alike our findings, they discovered that Attitudes Toward Robots influenced the sensitivity to the Uncanny valley.

### Occupation acceptability

We wondered how our uneasiness might affect our views on a robot having a job and working in contact with us. Our results indicated that only the *Attractiveness* is a good predictor of the Occupation acceptability and *Perceived Humanness* also affects it. The uneasiness felt by the participants did not affect their acceptance of the robot. This result is unexpected as one would think that the uneasiness would lead to rejection regardless of human-likeness and attractiveness of the robot. Here, the “*what is beautiful is good*” stereotype might to overcome the Uncanny effect of the robot and its motions. This stereotype supposes that beauty is strongly related to goodness, therefore good looking persons are better than less attractive persons. This cognitive bias is demonstrated by several studies (Eagly et al., 1991; Agthe et al., 2011). Researchers report that this bias was mostly true when the attractive person to be rated and the evaluator were of opposite sex. When both were of the same sex, the evaluator would feel threatened and then rated lower the attractive person (Agthe et al., 2011). In our study, participants who rated the robot attractive tend to see it working among us, without any effect of its *Eeriness*. Also, since the robot is by design sexless and does not possess any recognizable sexual attribute, positive bias might only apply. This result might be useful for robot designers wanting to overcome Uncanny valley phenomenon. We also found that Perceived Humanness had some influence on the occupation acceptability. Appearance of robots being a predictor on the occupation was studied by Hegel et al. (2009). They found that humanoid robots were thought more fit for occupations similar to humans and animal-like robots were thought adequate as pets or companions. This finding is along the lines of ours: the more the robot would be perceived as human, the more it will be seen fit for work.

## Conclusion

The Uncanny valley is an intriguing and not well-understood phenomenon. As robotics advances and world population ages, robots will be seen more and more in our daily life. Their behavior, their motions, their emotions might appear alien and thus provoke rejection and uneasiness from users. We propose to study what factors would influence users' impression of the robot. One unique robot, WABIAN-2R, was used for the experiment and only its motions changed, depending on the emotion and the emotional intensity.

One interesting result of this work is that we confirmed that the Uncanny valley to be a highly subjective matter. For the same humanoid robot, some participants perceived it as not quite human and some others found it very human-like. By plotting the participants' reactions to the robot emotional motions, we found the Uncanny valley. Nonetheless, our valley is not much related to the steep depression predicted by Mori when a machine is moving. It was rather a smoother valley similar to his predicted valley describing the still condition. The *Attitude toward robots* was the main influence of the Uncanny valley feeling. Participants who had positive views toward robots rated our robot and its motions less eerie and more attractive than those with negative views.

Another intriguing result is that the perceived *Attractiveness* of the robot had a major effect on its occupation acceptability regardless of how eerie it was rated. Also, the more human like, With a carefully planned external design, it would be possible to minimize any Uncanny valley phenomenon due to strange motions or behavior.

It would be interesting to reproduce the experiment with humanoid robots similar in shape such as ASIMO (Sakagami et al., 2002), HRP-2 (Hirukawa et al., 2004), or even ATLAS from Boston Dynamics. One limitation of the study is the use of videos as stimuli. Videos are useful to understand indirect interaction and impressions from perception. To understand what effect embodiment has, it is necessary to conduct real interaction with users. This will be the next step for our work.

### Conflict of interest statement

The authors declare that the research was conducted in the absence of any commercial or financial relationships that could be construed as a potential conflict of interest.
