# Supplementary material for: Walking in the uncanny valley: importance of the attractiveness on the acceptance of a robot as a working partner
Source: Front Psychol. 2015 Feb 25;6:204. doi: 10.3389/fpsyg.2015.00204 (PMC4340147; doi:10.3389/fpsyg.2015.00204)
Supplement: Supplementary file 2 [file DataSheet1.DOCX]

***Supplementary Material***

**Walking in the Uncanny Valley: Importance of the Attractiveness on the Acceptance of a Robot as a Working Partner**

**Matthieu Destephe^1^*, Martim Brandao^2^, Tatsuhiro Kishi^2^, Massimiliano Zecca^3^, Kenji Hashimoto^1^, Atsuo Takanishi^1,4^**

^1^Department of Modern Mechanical Engineering, Waseda University, Tokyo, Japan ^2^Graduate School of Science and Engineering, Waseda University, Tokyo, Japan
^3^School of Electronic, Electrical and Systems Engineering, Loughborough University, United Kingdom
^4^Humanoid Robotics Institute, Waseda University, Tokyo, Japan

*** Correspondence:** Matthieu Destephe, Department of Modern Mechanical Engineering, Waseda University, Japan.

contact@takanishi.mech.waseda.ac.jp

1. **Questionnaire**

This is the English translation of the questionnaire used for our work. The formatting style may vary from the online questionnaire we gave. We added comments in italics.

The questionnaire is divided into four parts:

### Part 1: General information

Sex

Age

Nationality

Education level

- Less than high school
- High-school diploma
- Associate degree
- Bachelor degree
- Master degree
- PhD degree or more

Current occupation

### Part 2: Questions about your experience with robots

**Robot-related experiences (6-point scale) 0, 1, 2, 3, 4, 5 or more**

1. How many times in the past one (1) year have you read robot-related stories, comics, news articles, product descriptions, conference papers, journal papers, blogs, or other material? How many times in the past one (1) year have you watched robot-related programs on ﬁlm, television, DVD, the Internet, or other media?
2. How many times in the past ten (10) years have you had physical contact with a robot?
3. How many times in the past ten (10) years have you attended robot-related lectures, exhibitions, trade shows, competitions, or other events?
4. How many times in your life have you built or programmed a robot?

**Attitudes toward robots** (7-point scale)

1. Select the statement that best describes your opinion.

- I strongly prefer people to robots. (-3)
- I strongly prefer robots to people. (+3)

1. Rate how warm or cold you feel toward robots.

- Very cold (-3)
- Very warm (+3)

1. Rate how warm or cold you feel toward people.

- Very cold (-3)
- Very warm (+3)

1. Select the statement that best describes your opinion.

- Robots are much more threatening than people. (+3)
- People are much more threatening than robots. (-3)

1. Rate how safe or threatening you feel robots are.

- Very safe (-3)
- Very threatening (+3)

1. Rate how safe or threatening you feel people are.

- Very safe (-3)
- Very threatening (+3)

1. How familiar are you with robots?

- Not at all familiar (-3)
- Completely familiar (+3)

1. How interested are you in robots?

- Not at all interested (-3)
- Really interested (+3)

*This questionnaire about the participant's robot-related experiences and their attitude towards robots based on the MacDorman questionnaire (MacDorman et al., 2008).*

### Part 3: General personality questions

Scale: Strongly agree, Slightly agree, Slightly disagree, Strongly disagree

1. I often notice small sounds when others do not.
2. I usually concentrate more on the whole picture, rather than the small details.
3. I find it easy to do more than one thing at once.
4. If there is an interruption, I can switch back to what I was doing very quickly.
5. I find it easy to 'read between the lines' when someone is talking to me.
6. I know how to tell if someone listening to me is getting bored.
7. When I'm reading a story I find it difficult to work out the characters' intentions.
8. I like to collect information about categories of things (e.g. types of car, types of bird, types of train, types of plant etc).
9. I find it easy to work out what someone is thinking or feeling just by looking at their face.
10. I find it difficult to work out people's intentions.

*This questionnaire is a short screening questionnaire for autism called AQ10 (Autism spectrum Quotient with 10 items) (Allison et al., 2012).*

# Survey introduction

This is the normal emotionless walking robot. Please watch it walking.

Normal walking (without emotion)

Video

# Survey

This a video of the robot walking with emotion.

After seeing this video, please answer the following questions.

Video

What do you think the robot expressed as emotion?

Anger

Happiness

Sadness

No emotion

Other

**In what kind of environment and place the movements and the emotions of the robot would be the most relevant?**

School

Law enforcement, police

Office reception

Hospital

Nowhere

**Please rate the robot and its walking.**

**What do you think about the movements of the robot?**

Artiﬁcial–Natural

Synthetic–Real

Inanimate–Living

Human-made–Humanlike

Mechanical Movement–Biological Movement

Without Deﬁnite Lifespan–Mortal

**What are your feelings about the robot?**

Reassuring–Eerie

Numbing–Freaky

Ordinary–Supernatural

Uninspiring–Spine-tingling

Boring–Shocking

Predictable–Thrilling

Bland–Uncanny

Unemotional–Hair-raising

**What do you think of the robot’s appearance?**

Unattractive–Attractive

Ugly–Beautiful

Repulsive–Agreeable

Crude–Stylish

Messy–Sleek

*This questionnaire assesses the participant's reactions and feelings about our emotional robot based on Ho's questionnaire (Ho & MacDorman, 2010) designed to assess the Uncanny valley phenomenon*.

1. **References**

Allison C., Auyeung B., Baron-Cohen S. (2012), Toward Brief “Red Flags” for Autism Screening: The Short Autism Spectrum Quotient and the Short Quantitative Checklist in 1,000 Cases and 3,000 Controls, Journal of the American Academy of Child and Adolescent Psychiatry 51(2), 202-212.

Ho CC, MacDorman KF. (2010), Revisiting the uncanny valley theory: Developing and validating an alternative to the Godspeed indices. Comput. Hum. Behav. 26(6), 1508-1518.

MacDorman KF, Vasudevan SK, Ho CC. (2008), Does Japan really have robot mania? Comparing attitudes by implicit and explicit measures. AI Soc. 23(4), 485-510.
